# Pathogenesis of Adenomyosis: An Integrated Review of Cellular Origins, Molecular Mechanisms, and Intersecting Diseases

**DOI:** 10.1111/jcmm.71129

**Published:** 2026-04-12

**Authors:** Jiang Yang, Xiaochong Li, Yu Peng, Zhongfeng Sun, Lingli Zhang, Jie Duan, Nanbert Zhong

**Affiliations:** ^1^ Department of Gynecology Maternal and Child Health Hospital of Hubei Province Wuhan China; ^2^ New York State Institute for Basic Research in Developmental Disabilities Staten Island New York USA

**Keywords:** adenomyosis, angiogenesis, epithelial–mesenchymal transition, immune dysfunction, stromal–epithelial crosstalk

## Abstract

Adenomyosis is a prevalent disorder of the archimetra, historically conflated with endometriosis but possessing a unique pathobiological trajectory. This review synthesises current molecular evidence to propose a unified mechanistic framework initiated by tissue injury and repair (TIAR), aberrant stem cell activation, or de novo metaplasia, all of which converge to disrupt the endometrial–myometrial interface disruption (EMID). Following this structural breach, a self‐perpetuating ‘vicious cycle’ is triggered, driven by four interconnected axes: (1) Hormonal Dysregulation, characterised by local hyper‐estrogenism, progesterone resistance, and a specific paracrine prolactin loop that drives reactive myometrial hypertrophy; (2) Immune‐Hemostatic Crosstalk, where activated platelets and M2‐polarised macrophages establish a pro‐fibrotic niche via TGF‐β1 signalling; (3) Hypoxia and Neuroangiogenesis, where HIF‐1α stabilisation orchestrates metabolic reprogramming (Warburg effect) and the pathological sprouting of sensory nerves, underpinning chronic pain; and (4) Epigenetic Fibrosis, driven by the oestrogen–slug–VEGF axis and HDAC‐mediated chromatin remodelling leading to epithelial–mesenchymal transition (EMT). Furthermore, we clarify the genetic distinction between adenomyosis and uterine leiomyomas, highlighting their divergent responses to androgen receptor signalling. By elucidating these molecular targets, we discuss emerging non‐hormonal therapeutics—including anti‐platelet agents and dopamine agonists—offering mechanism‐based strategies for fertility preservation.

**Abbreviations**
α‐SMA: alpha‐smooth muscle actin
AR: androgen receptor
ARID1A: AT‐rich interaction domain 1A
ATP: adenosine triphosphate
AUB: abnormal uterine bleeding
BDNF: brain‐derived neurotrophic factor
CDH1: cadherin‐1 (e‐cadherin)
CGRP: calcitonin gene‐related peptide
COX‐2: cyclooxygenase‐2
CTLs: cytotoxic T lymphocytes
DRD2: dopamine receptor D2
ECM: extracellular matrix
EGCG: epigallocatechin‐3‐gallate
EMI: Endometrial–Myometrial Interface
EMID: endometrial–myometrial interface disruption
eMSCs: endometrial mesenchymal stem‐like cells
EMT: epithelial‐to‐mesenchymal transition
EMT‐TFs: EMT‐inducing transcription factors
ERα: oestrogen receptor alpha
FAK: focal adhesion kinase
FMT: fibroblast‐to‐myofibroblast transdifferentiation
Gal‐9: galectin‐9
GLUT1: glucose transporter 1
HDAC: histone deacetylase
HIF‐1α: hypoxia‐inducible factor 1‐alpha
HK2: hexokinase 2
HLA‐G: human leukocyte antigen‐g
HMGCR: 3‐hydroxy‐3‐methylglutaryl‐coenzyme A reductase
IFN‐γ: interferon‐gamma
IL: interleukin (e.g., IL‐10, IL‐1β)
ILT2: immunoglobulin‐like transcript 2
KIR2DL4: killer cell immunoglobulin‐like receptor 2DL4
KRAS: kirsten rat sarcoma viral oncogene homologuehomolog
LDHA: lactate dehydrogenase a
lncRNA: long non‐coding RNA
LOX: lysyl oxidase
MCP‐1: monocyte chemoattractant protein‐1
MED12: mediator complex subunit 12
miRNA: micro‐RNA
MMP: matrix metalloproteinase (e.g., MMP‐2, MMP‐9)
mTORC1: mechanistic target of rapamycin complex 1
MVD: micro‐vessel density
NGF: nerve growth factor
NK (cells): natural killer cells
OS: oxidative stress
OT: oxytocin
OTR: oxytocin receptor
PDH: pyruvate dehydrogenase
PDK1: pyruvate dehydrogenase kinase 1
PDOs: patient‐derived organoids
PGE2: prostaglandin E2
PI3K: phosphoinositide 3‐kinase
PIK3CA: phosphatidylinositol‐4,5‐bisphosphate 3‐kinase catalytic subunit alpha
PKM2: pyruvate kinase M2
PR: progesterone receptor
PRL: prolactin
PRLR: prolactin receptor
ROS: reactive oxygen species
scRNA‐seq: single‐cell RNA sequencing
SIRT1: sirtuin‐1
SNAI1/2: snail family transcriptional repressor 1/2
SP: substance p
TCA cycle: tricarboxylic acid cycle
TCR: T cell receptor
TGF‐β1: transforming growth factor‐beta 1
TIAR: tissue injury and repair
TIM‐3: T cell immunoglobulin and mucin domain‐containing molecule 3
TKI: tyrosine kinase inhibitor
Treg: regulatory t cell
TRPV1: transient receptor potential vanilloid 1
TUG1: taurine‐upregulated gene 1
VEGF: vascular endothelial growth factor
VEGFR‐2: vascular endothelial growth factor receptor 2

## 1 Introduction

Adenomyosis is a prevalent and debilitating gynaecological disorder characterised by the benign invasion of endometrial glands and stroma into the myometrium. This ectopic presence induces a reactive response in the surrounding tissue, manifesting as myometrial hypertrophy, hyperplasia, and diffuse fibrosis [1]. Clinically, it presents a significant burden, causing severe dysmenorrhea, abnormal uterine bleeding (AUB), and infertility, often overlapping with endometriosis and uterine fibroids. While historically described as ‘endometriosis interna’ [2], adenomyosis is now recognised as a distinct entity originating from the dysfunction of the endometrial–myometrial interface (EMI), also known as the archimetra or junctional zone (JZ) [3, 4].

The true prevalence of adenomyosis has long been obscured by selection bias, as historical incidence rates were derived exclusively from hysterectomy specimens. A seminal study by Bird et al. demonstrated that detection rates are highly methodological, fluctuating between 31% and 61.5% depending on the number of histologic sections analysed [5]. However, the advent of high‐resolution non‐invasive imaging, including transvaginal ultrasound (TVUS) and magnetic resonance imaging (MRI), has revolutionised diagnosis. Recent epidemiological data indicate a prevalence of 20%–30% even in asymptomatic populations and, crucially, highlight an increasing diagnosis rate among adolescents and young women seeking fertility preservation [6, 7, 8].

Histopathologically, adenomyosis is defined by the invasion of endometrial tissue more than 2.5 mm beyond the endometrial–myometrial junction [9]. Unlike uterine leiomyomas, which form distinct, encapsulated nodules, adenomyosis is characterised by an infiltrative growth pattern. The lesion is surrounded by hypertrophic smooth muscle cells intermixed with normal myometrium, creating a diffuse pathology with no clear boundary [10]. This structural alteration is not merely anatomical but represents a profound disruption of the archimetral unit [3].

Despite its prevalence, the pathogenesis of adenomyosis remains complex. Traditional theories focusing solely on hyper‐estrogenism fail to explain the fibrotic and neuroangiogenic features of the disease. Consequently, current treatments are largely limited to hormonal suppression or hysterectomy, which are unsuitable for patients desiring pregnancy. Therefore, this review aims to synthesise current molecular evidence to propose a unified mechanistic framework. We will explore the origins of the disease and how a vicious cycle of immune‐hemostatic dysregulation, hypoxia‐driven neuroangiogenesis, and epigenetic fibrosis perpetuates the lesion, identifying novel non‐hormonal targets for therapeutic intervention.

## 2 Methods

To provide a comprehensive overview of the pathogenesis of adenomyosis, we conducted a systematic literature search using PubMed, Embase and Web of Science databases for articles published from inception up to December 2025. The search strategy combined Medical Subject Headings (MeSH) terms and free‐text keywords, including ‘adenomyosis’, ‘pathogenesis’, ‘aetiology’, ‘invagination’, ‘metaplasia’, ‘stem cells’, ‘epithelial–mesenchymal transition (EMT)’, ‘single‐cell RNA sequencing’ and ‘organoids’.

### 2.1 Inclusion and Exclusion Criteria

We prioritised studies that provided mechanistic insights into cellular origins, molecular pathways and genetic/epigenetic alterations. Given the rapid advancement in this field, special attention was given to high‐impact original research and reviews published within the last 5 years (2020–2025), particularly those utilising multi‐omics approaches. We also included seminal historical papers to contextualise the classical theories (e.g., TIAR theory). Only articles published in English were considered. The relevance of retrieved citations was assessed by screening titles and abstracts, followed by a full‐text review of selected papers to ensure they specifically addressed the molecular or cellular mechanisms of adenomyosis rather than solely clinical management.

## 3 Etiological Theories and Initiating Mechanisms

The precise etiopathogenesis of adenomyosis remains one of the most debated subjects in gynaecology. Historically viewed through a singular lens, contemporary understanding posits that adenomyosis is not a monolithic entity but rather a heterogeneous disorder arising from diverse yet interconnected cellular origins. While no single model fully captures the spectrum of disease manifestations, three canonical theories have emerged as the primary frameworks for understanding the genesis of ectopic lesions: (a) The Invagination and TIAR Theory, which attributes lesion formation to mechanical trauma at the EMI; (b) The Metaplasia of Embryonic Remnants Theory, which proposes that de novo lesions arise from misplaced Müllerian ducts; (c) The Stem Cell Origin Theory, which focuses on the aberrant migration and differentiation of adult endometrial or bone marrow‐derived progenitor cells.

Crucially, these theories are not mutually exclusive; rather, they represent distinct mechanistic pathways that often converge, ultimately leading to the structural disruption of the archimetra and the establishment of the adenomyotic micro‐environment.

### 3.1 Invagination and Tissue Injury/Repair (TIAR) Theory

The TIAR theory suggests that adenomyosis originates from the inward migration of endometrial tissue, beginning at the basalis layer, crossing the EMI, and embedding within the myometrium [11]. This theory challenges the notion of passive infiltration, suggesting instead that adenomyosis results from a reactive process driven by chronic auto‐traumatisation of the uterus.

The EMI serves as the principal site of uterine contractility in non‐pregnant women and plays a pivotal role in menstruation and reproductive processes such as sperm transport, implantation, and early pregnancy maintenance [12]. The uterus exhibits hyper‐peristalsis (increased frequency and amplitude of contractions) and elevated resting pressure, exerting significant mechanical shearing forces at the EMI [13] Chronic mechanical strain causes repetitive micro‐trauma or micro‐dehiscence at the basal aspect of the endometrial glands. This physical injury activates the localised TIAR system. Consequently, the physiological repair mechanism is overwhelmed, leading to the invagination of basal endometrial glands and stroma into the myometrial cracks as a maladaptive healing response.

Oestrogen is central to this process. It enhances oxytocin receptor (OTR) expression and increases myometrial contractility, fuelling a positive feedback loop in which trauma‐induced local hyper‐estrogenism further exacerbates hyper‐peristalsis via the TIAR mechanism [3, 4]. Mechanical strain simultaneously activates inflammatory pathways—most notably cyclooxygenase‐2 (COX‐2) and prostaglandin E2 (PGE2)—that up‐regulate steroidogenic acute regulatory protein (STAR) and P450 aromatase [14]. These enzymes, through oestrogen receptor‐beta (ER‐β) signalling, promote angiogenesis, fibroblast proliferation and chronic inflammation, ultimately impairing normal tissue repair [15].

Consequently, basal endometrial glands and stroma infiltrate the myometrium through micro‐structural gaps formed by repeated injury, establishing adenomyotic lesions. Over time, even physiological uterine contractions (normoperistalsis) may contribute to cumulative micro‐trauma, particularly in premenopausal women, potentially leading to late onset adenomyosis [16].

### 3.2 Metaplasia Theory

The metaplasia theory proposes that adenomyotic lesions can arise de novo through the metaplastic transformation of displaced embryonic pluripotent Müllerian remnants [13]. During embryonic development, the Müllerian ducts form the female reproductive tract, but residual pluripotent cells may remain embedded within the uterine wall. These cells retain the capacity to differentiate into endometrial glands and stroma under certain physiological or pathological conditions, potentially giving rise to ectopic endometrial tissue characteristic of adenomyosis.

Support for this theory comes from rare but compelling clinical observations. For instance, cases of adenomyosis have been reported in patients with Mayer–Rokitansky–Küster–Hauser (MRKH) syndrome, a congenital condition characterised by the absence of functional endometrial tissue [17, 18]. Despite the lack of native endometrium, adenomyotic nodules have been identified in the rudimentary uterine musculature of these individuals—strongly implicating a non‐endometrial, embryologic origin for the ectopic tissue.

This hypothesis broadens the understanding of adenomyosis origins, suggesting that not all lesions necessarily result from the eutopic endometrium but may instead develop from aberrant differentiation of embryonic cell rests.

### 3.3 Stem Cell Theory

The stem cell theory advances our understanding of adenomyosis pathogenesis by shifting the focus from the passive displacement of mature tissue to the aberrant migration and differentiation of adult stem cells. This model effectively addresses the limitations of the classical invagination theory, offering a compelling explanation for the high proliferative capacity, persistence, and recurrence potential of ectopic lesions.

Current evidence points to two primary cellular origins. The predominant source is the endometrial basalis layer [19, 20]. Recent 3D‐reconstructions have identified a protected niche of epithelial progenitor cells within the horizontal glands of the basalis [21]. Upon micro‐trauma or structural disruption of the EMI, these basalis‐resident progenitors are hypothesised to escape their niche [22]. A secondary, albeit less dominant, source involves bone marrow‐derived stem cells (BMDSCs) [23]. Evidence from HLA‐mismatched transplants demonstrates that circulating BMDSCs can be recruited to the ischemic or injured uterus, where the pathological micro‐environment drives their maladaptive differentiation into ectopic stromal and glandular components [24].

Advancements in single‐cell RNA sequencing (scRNA‐seq) have provided unprecedented resolution of these pathogenic progenitors. Histologically observed as undifferentiated ‘pale cells’ at the endometrial–myometrial junction, these cells are now molecularly characterised by distinct stemness profiles [25]. Specifically, a regenerative SOX9+/LGR5+ epithelial progenitor population has been identified as a critical reservoir for endometrial regeneration [26, 27, 28]. In the context of adenomyosis, these specific progenitors exhibit an up‐regulation of survival and stemness pathways (such as Notch1 and c‐Kit) alongside a concurrent loss of cell adhesion molecules like E‐cadherin [29, 30]. This molecular profile—combining high regenerative potential with increased motility—pinpoints them as the primary drivers of myometrial invasion.

The transformation of these progenitors into frank adenomyotic lesions is a dynamic, multi‐step cascade. Triggered by chronic inflammation or mechanical strain (TIAR), quiescent basalis progenitors are activated and undergo EMT [31, 32]. This phenotypic switch grants them the invasive capacity required to breach the myometrium. Once embedded in the ectopic niche, local growth factors drive their re‐differentiation into functional endometrial glands and stroma. Crucially, because these rogue cells originate from the basalis layer, the resulting lesions inherently retain ‘basalis‐like’ traits—most notably, profound progesterone resistance and robust proliferative capacity [3, 33] —which intrinsically perpetuate the disease course.

### 3.4 Integrating the Theories: The EMID Convergence

While historically viewed as competing hypotheses, the TIAR, stem cell and metaplasia theories are not mutually exclusive; rather, they functionally converge on the endometrial–myometrial interface disruption (EMID) [4]. EMID should not be conceptualised solely as a mechanical tear. Instead, it is a bi‐directional vulnerability. From a top‐down mechanical perspective, hyper‐peristalsis (TIAR) inflicts direct physical micro‐trauma on the junctional zone. Simultaneously, from a bottom‐up biochemical perspective, aberrantly activated endometrial mesenchymal stem‐like cells (eMSCs) residing in the basalis layer undergo EMT [34]. These hyper‐active progenitor cells secrete elevated levels of matrix metalloproteinases (specifically MMP‐2 and MMP‐9), actively and enzymatically degrading the basement membrane of the EMI in situ [13]. Furthermore, local metaplasia of Müllerian remnants within the myometrium creates distinct inflammatory micro‐niches that further compromise the structural integrity of the surrounding smooth muscle [34]. Thus, mechanical strain and aberrant stem‐cell‐driven enzymatic degradation act synergistically to permanently breach the EMID, permitting full‐scale invagination.

## 4 Pathogenetic Mechanisms

### 4.1 Hormonal Dysregulation

Once the physical barrier of the archimetra is breached, the survival and expansion of ectopic lesions are immediately fuelled by a profound hormonal imbalance. This dysregulation is not merely a reflection of systemic ovarian function but the result of complex epigenetic reprogramming and paracrine failure within the local micro‐environment.

#### 4.1.1 Local Oestrogen Biosynthesis and Epigenetic Reprogramming

In adenomyosis, oestrogen is the primary driver of ectopic tissue proliferation. Beyond mere growth, hyper‐estrogenism directly induces uterine hyper‐peristalsis (exacerbating the mechanical TIAR mechanism) [3] and stimulates the production of pro‐inflammatory cytokines, creating a highly vascularised and inflamed micro‐environment [4].

The fierce hyper‐estrogenic micro‐environment within adenomyotic lesions is driven by profound epigenetic alterations governing oestrogen metabolism and receptor expression. The de novo local synthesis of oestrogen is primarily driven by steroidogenic factor‐1 (SF‐1) [35]. In the healthy endometrium, SF‐1 is silenced by MeCP2; however, in adenomyosis, severe hypo‐methylation of the SF‐1 promoter releases this inhibition. Activated SF‐1 synergises with USF2 and GATA6 to massively up‐regulate aromatase (CYP19A1) and steroid sulfatase (STS). Concurrently, local oestrogen inactivation is catastrophically impaired. The gene encoding 17β‐hydroxysteroid dehydrogenase type 2 (17β‐HSD2) undergoes dense DNA hyper‐methylation, leading to the complete silencing of the enzyme responsible for converting highly active oestradiol (E2) into the weaker estrone (E1). This results in a toxic local accumulation of E2 [36].

Oestrogen receptor signalling also undergoes an epigenetic inversion. Hypo‐methylation of the ESR2 gene leads to the dramatic overexpression of oestrogen receptor beta (ERβ). This overexpression not only suppresses ERα (contributing to progesterone resistance) but directly activates the NLRP3 inflammasome via caspase‐1. The subsequent massive release of highly pro‐inflammatory IL‐1β fuels the COX‐2/PGE2/aromatase positive feedback loop, perfectly intertwining hormonal hyper‐activation with chronic inflammation [37, 38].

#### 4.1.2 Progesterone Resistance: Epigenetic and Paracrine Failure

In normal physiology, progesterone counteracts oestrogen to halt proliferation and induce decidualisation. In adenomyosis, the ectopic tissue exhibits profound ‘progesterone resistance’. This failure leaves the oestrogen‐driven proliferation entirely unopposed and prevents normal stromal‐epithelial crosstalk, resulting in a fragile, bleeding‐prone architecture.

Progesterone resistance in adenomyosis extends beyond the simple loss of a single receptor; it represents the complete collapse of the paracrine and anti‐fibrotic networks. The profound down‐regulation of progesterone receptor isoform B (PR‐B) originates from the hyper‐methylation of the PGR promoter [39]. Furthermore, recent evidence indicates that frequent somatic KRAS mutations in the ectopic epithelium hyper‐activate downstream MAPK signalling, which epigenetically locks down PR expression [40]. Progesterone resistance directly results in the loss of Krüppel‐like factor 11 (KLF11), a critical transcriptional repressor. The absence of KLF11 unleashes pro‐fibrotic targets such as COL1A1 and TGF‐β, removing physiological restraints on collagen deposition and leading to irreversible, diffuse myometrial fibrosis [41, 42].

The failure of stromal cells to undergo decidualisation (marked by the down‐regulation of FOXO1 and HOXA10) renders the local architecture fragile and prone to micro‐bleeding. Crucially, the precipitous drop in paracrine inhibitory factors like Glycodelin (PAEP) and CXCL14 severs normal stromal‐epithelial crosstalk, allowing the ectopic glands to maintain high oestrogen responsiveness and extreme invasiveness throughout the menstrual cycle [27].

#### 4.1.3 The Prolactin (PRL) Axis and Single‐Cell Revelations

Historically, PRL has been recognised as a locally synthesised autocrine/paracrine growth factor in the uterus [43]. However, recent landmark research, culminating in high‐resolution single‐cell transcriptomic atlases, has elevated PRL from a secondary player to the primary pathogenic driver of adenomyosis [44, 45]. The characteristic diffuse, globular enlargement of the adenomyotic uterus is not merely a passive reaction, but a highly orchestrated remodelling process driven by the massive local secretion of PRL from ectopic endometrial glands.

Rather than acting on a single cell type, advanced scRNA‐seq reveals that this hyper‐active PRL signalling operates through a coordinated, multi‐target network. Ectopic epithelial cells, particularly a distinct ‘ECM‐high’ sub‐population caught in an active state of EMT, intensely overexpress prolactin receptors (PRLR). In this niche, high local PRL concentrations exert potent anti‐apoptotic effects, granting these rogue cells a formidable survival advantage as they infiltrate the rigid myometrium [8]. PRL profoundly disrupts the stromal micro‐environment. On one hand, it functionally compromises normal basalis fibroblasts (Fib_C7), physically ‘softening’ the EMI barrier to permit unimpeded invasion. On the other hand, PRL actively stimulates an inflammatory fibroblast sub‐cluster (‘ds1’), driving their rapid trans‐differentiation into myofibroblasts. This PRL‐induced deposition of cross‐linked collagen and pro‐inflammatory cytokines is the true structural engine for diffuse uterine fibrosis and severe dysmenorrhea [8]. Simultaneously, PRL acts on adjacent smooth muscle cells, acting as a powerful mitogen that drives the robust myometrial hypertrophy classically observed in clinical presentations [45].

By mapping this comprehensive PRL/PRLR network, researchers have definitively validated PRL as a master therapeutic target. Transgenic overexpression of PRL or pituitary transplantation in animal models is sufficient to directly induce the adenomyosis phenotype, an effect that is completely reversible by dopamine agonists [46, 47]. Most importantly, targeted blockade of this axis utilising monoclonal antibodies (e.g., HMI‐115 against PRLR) has been shown to markedly ameliorate these multi‐cellular pathological manifestations, establishing PRL inhibition as one of the most promising non‐hormonal, fertility‐sparing therapeutic strategies currently in development [48].

#### 4.1.4 The OTR and Uterine Hyper‐Peristalsis

While steroid hormones fuel cellular proliferation in adenomyosis, the peptide hormone oxytocin (OT) provides the mechanical force driving disease progression. OT induces hyper‐peristalsis and dysperistalsis within the junctional zone (archimetra). These violent, uncoordinated contractions inflict recurrent physical micro‐trauma at the EMI. This profound mechanical strain directly initiates the TIAR mechanism, physically driving basal endometrial cells deep into the traumatised myometrium [49].

The hyper‐sensitivity of the adenomyotic uterus to OT arises not from systemic OT elevation, but from a profound local overexpression of OTRs in both the eutopic endometrium and surrounding myometrium [50]. Crucially, this overexpression is synergistically amplified by the local hyper‐estrogenic micro‐environment: oestrogen directly up‐regulates OTR transcription via oestrogen receptor alpha (ERα). Consequently, a self‐perpetuating vicious cycle is established: elevated local oestrogen → OTR up‐regulation → severe hyper‐peristalsis → mechanical EMI disruption (TIAR) → exacerbated inflammation and sustained oestrogen synthesis [4].

Clinically, the hyper‐activation of the OT/OTR axis underpins the hallmark severe dysmenorrhea and reproductive failure seen in patients. Abnormal uterine contractions disrupt embryo implantation, contributing to infertility and early miscarriage. Consequently, targeting the OT signalling pathway represents a highly promising therapeutic frontier. Recent clinical evidence demonstrates that administering OTR antagonists (e.g., Atosiban) during assisted reproductive technology (ART) cycles, such as frozen–thawed embryo transfer (FET), effectively suppresses uterine hyper‐peristalsis and significantly improves pregnancy outcomes while reducing early miscarriage rates in patients with adenomyosis. This functionally corroborates the central role of OT in structural and reproductive disruption [51].

### 4.2 Chronic Inflammation and Immune‐Hemostatic Crosstalk: The Establishment of Immune Privilege

The persistence of adenomyotic lesions is contingent upon the profound remodelling of the local immune micro‐environment. This process is characterised by a pathological transition from acute defence mechanisms to a state of sustained chronic inflammation coupled with immune tolerance. This transition is not a passive event but is orchestrated by a complex crosstalk between the coagulation cascade and the immune response.

#### 4.2.1 Platelet‐Mediated Initiation of Inflammatory Signalling

The TIAR theory posits that mechanical disruption at the EMI serves as the inciting event. This micro‐trauma triggers the immediate recruitment and activation of platelets [52]. Upon activation, platelets undergo degranulation, releasing a potent milieu of bioactive molecules, including transforming growth factor‐beta 1 (TGF‐β1), platelet‐derived growth factor (PDGF) and vascular endothelial growth factor (VEGF) [53, 54].

In the context of adenomyosis, platelets function beyond haemostasis as critical inflammatory amplifiers. Drawing upon established mechanisms of vascular inflammation [55], it is highly plausible that activated platelets express P‐selectin to form platelet‐leukocyte aggregates (PLAs) with circulating monocytes, a process hypothesised to actively facilitate the extravasation of immune cells into the dense myometrium. This process effectively translates physical injury into molecular chronic inflammation, maintaining the pro‐survival micro‐environment through the continuous activation of the NF‐κ B pathway and the COX‐2/PGE2 axis [56].

#### 4.2.2 Functional Reprogramming and Macrophage Polarisation

Macrophages dictate the survival of ectopic tissue. A distinct spatial dichotomy exists: the eutopic endometrium suffers from regulatory M2 depletion, whereas ectopic myometrial lesions recruit and reprogram a massive influx of macrophages [57, 58].

Driven by severe local hypoxia, high E2 concentrations, and platelet‐derived TGF‐β, infiltrating monocytes shift overwhelmingly toward an M2 (alternatively activated) phenotype [59, 60]. They secrete elevated levels of interleukin‐10 (IL‐10) and TGF‐β [61], effectively dampening T‐cell cytotoxicity and halting dendritic cell maturation. Unlike typical tumour‐associated macrophages (TAMs) that primarily drive angiogenesis, adenomyotic M2 macrophages exhibit exhaustive phagocytotic activity [60]. This efficiently clears cyclic haemorrhagic debris without triggering acute tissue necrosis, maintaining a ‘smouldering’ chronic inflammation that favours lesion survival [58].

#### 4.2.3 Molecular Mechanisms of Evasion From Immune Surveillance

Despite cyclic bleeding and cellular structural transitions, ectopic lesions successfully evade host natural killer (NK) cells and cytotoxic T lymphocytes (CTLs) by actively co‐opting physiological pathways of foetal tolerance and tumour evasion [62].

##### 4.2.3.1 Platelet cloaking and NK cell impairment

Cyclic injury triggers intense platelet aggregation, forming a physical ‘cloak’ that shields ectopic cells from immune recognition. Furthermore, platelet‐derived TGF‐β severely down‐modulates the NKG2D activating receptor on NK cells, rendering them functionally anergic and blind to the lesion [59, 60].

##### 4.2.3.2 HLA‐G Mediated Tolerance

Mimicking the maternal‐foetal interface, ectopic epithelial and stromal cells massively overexpress the non‐classical MHC molecule HLA‐G [63]. HLA‐G binds to inhibitory receptors (KIR2DL4 and ILT2) on both NK and T cells, completely blocking the release of perforin and granzyme, thereby halting cytolytic effector functions [64].

##### 4.2.3.3 The TIM‐3/Galectin‐9 Checkpoint Axis

Adaptive immune clearance is neutralised via the TIM‐3/Gal‐9 pathway, which is highly elevated in adenomyosis [65]. Lesion‐derived Gal‐9 binds to the TIM‐3 receptor on infiltrating T cells, disrupting TCR signalling [24]. This triggers a dual immunosuppressive effect: it drives cytotoxic T cells into terminal exhaustion and apoptosis, while simultaneously stimulating the proliferation of highly suppressive Regulatory T cells (Tregs) [65].

Collectively, the chronic inflammation ignited by platelet activation, combined with M2‐macrophage‐driven immunosuppression and HLA‐G‐mediated shielding, establishes an immune‐privileged niche that facilitates the implantation, survival, and progression of adenomyosis.

### 4.3 Hypoxia, Angiogenesis and Neurogenic Remodelling

The survival and expansion of ectopic endometrial tissue within the myometrium impose a significant metabolic demand. To sustain this growth, adenomyotic lesions must establish a dedicated vascular supply. This process is driven by a profound metabolic stress—hypoxia—which orchestrates a tripartite response: metabolic reprogramming, pathological angiogenesis, and neurogenesis.

#### 4.3.1 The Hypoxic Niche and Metabolic Reprogramming

Chronic tissue injury (TIAR) creates a fibrotic, poorly perfused micro‐environment characterised by severe hypoxia [11]. Hypoxia halts prolyl hydroxylase domain (PHD) enzymes, allowing HIF‐1α to escape degradation. HIF‐1α translocates to the nucleus to initiate a massive transcriptional cascade [66].

HIF‐1α forces a metabolic switch from oxidative phosphorylation to aerobic glycolysis for rapid ATP generation [67]. This is driven by the coordinated up‐regulation of GLUT1 (glucose uptake), HK2 (glucose trapping) and PDK1 (which inhibits the PDH complex, blocking mitochondrial TCA cycle entry). The up‐regulation of LDHA converts pyruvate into lactate. This acidic byproduct activates MMPs for tissue invasion and profoundly paralyses the cytotoxic functions of local NK cells and CD8+ T lymphocytes [67, 68]. Concurrently, the PI3K/Akt/mTOR pathway fuels lipid and nucleotide biosynthesis for rapid proliferation [69].

#### 4.3.2 Pathological Angiogenesis: The VEGF Cascade

The primary downstream effect of HIF‐1α is the up‐regulation of VEGF and its receptor VEGFR‐2 [70]. Unlike the orderly, cyclic angiogenesis of the eutopic endometrium, the vascular network in adenomyosis is chaotic and immature.

Adenomyotic neovasculature severely lacks pericyte coverage (CD31+/SMAα‐), rendering micro‐vessels fragile and chronically ‘leaky’ [71]. The vicious cycle of iron overload: continuous extravasation of erythrocytes leads to massive free iron release. This triggers the Fenton reaction and severe oxidative stress (OS) [72, 73]. High ROS levels physically disable PHD enzymes, permanently stabilising HIF‐1α. Concurrently, extravasated platelets release TGF‐β1 to drive dense myometrial fibrosis [74].

#### 4.3.3 Targeted Therapeutics: Recent Advances in Anti‐Angiogenic Strategies

Given the centrality of the hypoxic‐vascular axis, recent studies have explored non‐hormonal agents that disrupt these pathways. Emerging evidence highlights several promising candidates, which are comprehensively summarised in Table 1.

**TABLE 1:** Anti‐angiogenic targeted agents and molecular mechanisms in adenomyosis therapy.

Targeted therapeutic agent	Primary molecular target/mechanism	Downstream pathological interference	Preclinical/clinical validation
Cabergoline/Quinagolide [75, 76]	DRD2 Agonist	Induces VEGFR‐2 endocytosis; inhibits VEGF signalling	Normalises vasculature and reduces lesion MVD; high clinical safety
Ozagrel/Clopidogrel [77]	TXA2 Synthase/Receptor Inhibitor	Suppresses platelet activation; blocks TGF‐β1and VEGF release	Inhibits myometrial infiltration, EMT, and fibrogenesis
Axitinib [78]	VEGFR‐2 Tyrosine Kinase Inhibitor	Directly blocks VEGF cascade; inhibits angiogenesis	Significantly reduces disease severity index in murine models
Resveratrol [79]	SIRT1 Activator	Deacetylates HIF‐1α; suppresses hypoxic stress response	Reduces endometrial fibrosis and alleviates hyper‐algesia
Atorvastatin [80]	HMGCR Inhibitor	Inhibits Rho/Rac prenylation; exerts pleiotropic anti‐inflammatory effects	Reduces MMP‐9 and MCP‐1; limits macrophage recruitment

##### 4.3.3.1 Dopamine Agonists (e.g., Cabergoline, Bromocriptine)

Traditionally used for hyper‐prolactinemia, these agents have shown efficacy in shrinking adenomyotic lesions. Mechanistically, they activate dopamine receptor D2 (DRD2) on endothelial cells, promoting the endocytosis of VEGFR‐2, thereby desensitising the cells to VEGF stimulation and normalising vascular permeability [75, 76].

##### 4.3.3.2 Anti‐Platelet Therapy (e.g., Ozagrel, Clopidogrel)

Clopidogrel and Ozagrel (a thromboxane A2 synthase inhibitor) prevent platelet degranulation, definitively halting the release of TGF‐β1 and VEGF. Preclinical models demonstrate that this successfully starves the Smad‐signalling pathway, halting EMT and slowing myometrial fibrogenesis [77].

##### 4.3.3.3 Natural Compounds (e.g., Epigallocatechin‐3‐Gallate [EGCG], Resveratrol)

Recent investigations into phytochemicals have identified potent anti‐angiogenic properties. EGCG (green tea extract) has been shown to inhibit VEGF expression and suppress HIF‐1α accumulation in ectopic endometrial cells [81, 82]. Similarly, resveratrol activates Sirtuin‐1 (SIRT1) to destabilise HIF‐1α, offering a multi‐target approach to reduce vascularisation and fibrosis [79, 83, 84].

##### 4.3.3.4 Statins (e.g., Atorvastatin)

By inhibiting HMGCR and reducing the prenylation of pro‐inflammatory GTPases (Rho/Rac), statins exert profound pleiotropic effects. They massively down‐regulate local VEGF, CD31, MMP‐9 and MCP‐1 expression, physically interfering with neoangiogenesis and macrophage recruitment [80, 85].

### 4.4 Tissue Remodelling: The EMT‐FMT‐Fibrosis Cascade

The hallmark of adenomyosis is the profound structural remodelling of the uterine wall. This process is not a singular event but a continuous cascade: epithelial cells first acquire motility (EMT), invade the myometrium, and subsequently induce the surrounding stroma to form a fibrotic scar (FMT and Fibrosis) [86].

#### 4.4.1 The ‘Cadherin Switch’ and Partial EMT

Invasion at the EMI depends entirely on breaking epithelial homeostasis. Normally, epithelial cells are tethered by adherens junctions mediated by E‐cadherin (CDH1). The invasive phenotype is defined by the ‘Cadherin Switch’—the profound down‐regulation of E‐cadherin and compensatory up‐regulation of mesenchymal markers like N‐cadherin and Vimentin [4, 87].

This repression is orchestrated by pleiotropic EMT‐inducing transcription factors (EMT‐TFs), predominantly Snail (SNAI1) and Slug (SNAI2), alongside ZEB1/2 and TWIST [87]. However, rather than undergoing a complete binary transition into solitary mesenchymal cells, invading ectopic glands undergo a ‘Partial EMT’, facilitating collective cell migration [88, 89]. The leading front cells up‐regulate mesenchymal TFs (Snail/Slug) to acquire motility and matrix‐degrading capabilities; The follower cells maintain functional cell–cell contacts (via P‐cadherin and residual E‐cadherin) to preserve the distinct glandular architecture pathognomonic of adenomyosis. This hybrid state provides optimal biomechanical balance, allowing cells to navigate the physical stiffness gradient (durotaxis) into the dense myometrium while shielding inner cells from immune surveillance and anoikis [90, 91].

#### 4.4.2 Converging Signalling Pathways: The Drivers of Plasticity

The induction of this phenotypic switch is not random but orchestrated by a convergence of hormonal, inflammatory, and developmental signals.

##### 4.4.2.1 The Oestrogen‐Slug‐VEGF Axis

Hyper‐estrogenism drives EMT. Oestrogen receptor alpha (ERα) up‐regulates Slug (SNAI2). Slug acts as a dual pathogenic linchpin: it represses CDH1 to initiate partial EMT and acts as a potent transcriptional activator of VEGF [92, 93]. This perfectly couples tissue invasion with neoangiogenesis, ensuring the invading front has the necessary blood supply.

##### 4.4.2.2 The Platelet‐TGF‐β/Smad Loop

Chronic mechanical trauma (TIAR) induces platelet extravasation. Activated platelets release latent TGF‐β1. TGF‐β1 phosphorylates intracellular Smad2/3, which translocates to the nucleus to cooperate with Snail/Slug, driving persistent pro‐EMT and pro‐fibrotic stimuli [87, 94].

##### 4.4.2.3 The Wnt/β‐Catenin and TGF‐β2 Cycle

The loss of E‐cadherin liberates β‐catenin into the cytoplasm. Stabilised nuclear β‐catenin directly binds the TGFB2 promoter, inducing profound TGF‐β2 expression. Secreted TGF‐β2 then hyper‐activates Smad signalling, increasing Snail/Slug levels, which further represses E‐cadherin. This acts as a molecular ‘ratchet’, irreversibly locking cells into an aggressive mesenchymal state [93].

#### 4.4.3 Fibroblast‐to‐Myofibroblast Transdifferentiation (FMT) and Fibrosis

Once the ectopic tissue establishes itself in the myometrium, the pathological process shifts from invasion to stiffening. The persistent inflammatory milieu drives resident fibroblasts and EMT‐derived cells to undergo FMT [95].

These myofibroblasts (α‐SMA+) are hyper‐active secretory cells. Driven by the PI3K/Akt/mTOR axis—which is up‐regulated by the stiff fibrotic matrix itself (mechanotransduction)—they deposit excessive collagen I and fibronectin [96]. This deposition disrupts the normal myometrial architecture, leading to the characteristic ‘globular’ enlargement and biomechanical rigidity of the uterus.

#### 4.4.4 Epigenetic Locking: The Mechanism of Irreversibility

Crucially, the fibrotic phenotype in adenomyosis is often resistant to hormonal therapy due to epigenetic locking.

##### 4.4.4.1 Histone Modification

The recruitment of histone deacetylases (HDACs) by Snail/Slug to the E‐cadherin promoter leads to chromatin compaction and permanent gene silencing [93, 97].

##### 4.4.4.2 Non‐Coding RNAs

Dysregulation of non‐coding RNAs, such as the down‐regulation of miR‐145‐5p (which normally targets ZEB2) and the up‐regulation of lncRNA TUG1, further stabilises the invasive and fibrotic machinery [98, 99].

This epigenetic rigidity explains why fibrosis persists even after hormonal levels are suppressed, highlighting the potential utility of HDAC inhibitors (e.g., valproic acid) to ‘re‐open’ chromatin and reverse the disease phenotype [100].

## 5 Correlation With Uterine Comorbidities: Shared Soils, Divergent Seeds

Although adenomyosis, endometriosis and uterine leiomyomas frequently co‐occur (with co‐occurrence rates reaching 30%–50%) and share a highly overlapping clinical presentation of uterine enlargement and hormonally responsive pelvic pain, they represent fundamentally distinct pathogenic trajectories. Advanced molecular profiling allows for the precise segregation of these pathologies across three definitive axes: genetic clonality, spatial remodelling, and distinct hormonal receptor landscapes.

The genomic architecture of the lesion strictly defines its primary cellular origin and neoplastic potential. Leiomyomas are monoclonal tumours of the smooth muscle, with approximately 70% harbouring somatic mutations in mediator complex subunit 12 (MED12) [101, 102]. These mutations drive tumour proliferation through the Wnt/β‐catenin pathway. In contrast, adenomyosis is characterised by activation of the KRAS pathway in the epithelial component [40]. Crucially, the surrounding smooth muscle hypertrophy in adenomyosis lacks MED12 mutations, confirming it is a reactive process driven by paracrine factors rather than a primary neoplasm.

Adenomyosis represents an ‘Inside‐Out’ invasion driven by the TIAR mechanism and invagination of the basalis layer into the myometrium. Endometriosis is predominantly an ‘Outside‐In’ process driven by retrograde menstruation and the implantation of the functionalis layer onto peritoneal surfaces. The inflammatory response in adenomyosis uniquely induces diffuse, integral myometrial fibrosis via FMT, irrevocably altering the uterine architecture. While endometriotic lesions incite severe pelvic inflammation and dense external adhesions, they do not induce the massive, organ‐wide smooth muscle hyperplasia characteristic of the adenomyotic uterus.

The most profound divergence among these pathologies lies in their localised endocrine micro‐environments. The expression status of AR establishes a critical boundary between myometrial and endometrial‐derived pathologies. In leiomyomas, AR is frequently overexpressed [103]. Here, endogenous androgens function synergistically with oestrogen as mitogenic ‘fuel’, actively promoting tumour expansion. Conversely, in endometrium‐derived pathologies such as endometriosis, AR expression is frequently down‐regulated in the ectopic tissue [104]. Physiologically, androgen signalling in the endometrium acts as a critical ‘anti‐proliferative brake’—counteracting estrogenic drive and actively up‐regulating progesterone receptors (PRs) to maintain hormonal homeostasis [105]. The pathological loss of AR in ectopic lesions contributes directly to their uncontrolled, oestrogen‐driven expansion. This fundamental divergence beautifully explains their contrasting therapeutic responses [106]: administering androgenic agents (e.g., Danazol) exploits residual AR to forcibly re‐engage this ‘brake’ and induce lesion atrophy in endometriosis and adenomyosis, whereas such agents are strictly contraindicated for leiomyomas, where they would inadvertently fuel further tumour expansion.

## 6 Conclusion and Future Perspectives

Adenomyosis is a profoundly complex disorder of the archimetra, initiated by mechanical micro‐trauma (TIAR) and perpetuated by a vicious cycle of immune‐hemostatic dysregulation, pathological angiogenesis, and massive fibrotic remodelling. While it shares the ‘common soil’ of oestrogen dependence and progesterone resistance with leiomyomas and endometriosis, its distinct genetic drivers (e.g., epithelial KRAS vs. stromal MED12) and unique tissue micro‐environment firmly establish it as a singular pathological entity.

Despite profound mechanistic advancements, several critical translational bottlenecks currently impede the realisation of a clinical cure. Firstly, current research relies predominantly on end‐stage hysterectomy specimens representing burned‐out fibrotic disease. Elucidating the early initiation phase—specifically, how the very first endometrial cell breaches the myometrial barrier—remains the Holy Grail for preventative intervention. Secondly, adenomyosis encompasses highly heterogeneous clinical sub‐types (intrinsic vs. extrinsic, focal vs. diffuse). Chemically or hormonally induced murine models fail to capture this diversity, frequently causing therapies that succeed in mice to fail in diverse human populations. Thirdly, while highly effective anti‐fibrotic and epigenetic targets (e.g., HDAC and TGF‐β inhibitors) have been identified, their systemic administration poses unacceptable risks to ovarian function and endometrial receptivity in reproductive‐aged women.

To breach these barriers, future research must pivot decisively toward precision medicine and advanced bioengineering. Transitioning from conventional rodent models to patient‐derived organoids (PDOs) and ‘Organ‐on‐a‐Chip’ platforms. These advanced systems can accurately recreate the human‐specific EMI micro‐environment, enabling high‐throughput, personalised therapeutic screening. Prioritising the validation of non‐invasive biomarkers, such as circulating exosomal miRNAs associated with the KRAS or hypoxia/HIF‐1α pathways. This would enable definitive diagnosis and intervention long before the onset of irreversible, wood‐like fibrosis. Shifting the therapeutic paradigm away from broad hormonal suppression—which strictly precludes pregnancy—toward localised, uterus‐targeted delivery systems (e.g., nanomedicine). This will allow the safe deployment of downstream effectors like anti‐platelet agents, DRD2 agonists and epigenetic modulators directly to the myometrium without compromising systemic fertility.

The transition from viewing adenomyosis as a mysterious and elusive disease to a precisely defined disorder of archimetral remodelling opens unprecedented therapeutic avenues. The future of adenomyosis management lies not in the blunt instrument of hormonal suppression, but in the precise molecular interception of its localised immune, vascular, and fibrotic cascades.

## Author Contributions

**Xiaochong Li:** conceptualization, resources, data curation. **Lingli Zhang:** conceptualization. **Jiang Yang:** conceptualization, data curation, formal analysis, writing – review and editing, writing – original draft, resources, software. **Jie Duan:** conceptualization. **Yu Peng:** visualization, resources. **Zhongfeng Sun:** investigation.

## Funding

The funders had no role in the study design, data collection and analysis, decision to publish, or preparation of the manuscript.

## Conflicts of Interest

The authors declare no conflicts of interest.

## Data Availability

Data sharing is not applicable to this article as no new primary data were created or analysed in this study. All data and literature underlying this review are available within the article and its properly cited references.
